# Investigating the potential use of an Antarctic variant of *Janthinobacterium lividum* for tackling antimicrobial resistance in a One Health approach

**DOI:** 10.1038/s41598-018-33691-6

**Published:** 2018-10-15

**Authors:** Andreea Baricz, Adela Teban, Cecilia Maria Chiriac, Edina Szekeres, Anca Farkas, Maria Nica, Amalia Dascălu, Corina Oprișan, Paris Lavin, Cristian Coman

**Affiliations:** 1 NIRDBS, Institute of Biological Research, Cluj-Napoca, Romania; 2 Faculty of Biology and Geology, Babeş-Bolyai University, Cluj-Napoca, Romania; 3 Dr. V. Babeș Clinical Hospital of Infectious and Tropical Diseases Bucharest, Bucharest, Romania; 4 Carol Davila University of Medicine and Pharmacy, Bucharest, Romania; 5 Laboratorio de Complejidad Microbiana y Ecología Funcional, Instituto Antofagasta, Universidad de Antofagasta, Antofagasta, Chile

## Abstract

The aim of this paper is to describe a new variant of *Janthinobacterium lividum* - ROICE173, isolated from Antarctic snow, and to investigate the antimicrobial effect of the crude bacterial extract against 200 multi-drug resistant (MDR) bacteria of both clinical and environmental origin, displaying various antibiotic resistance patterns. ROICE173 is extremotolerant, grows at high pH (5.5–9.5), in high salinity (3%) and in the presence of different xenobiotic compounds and various antibiotics. The best violacein yield (4.59 ± 0.78 mg·g^−1^ wet biomass) was obtained at 22 °C, on R2 broth supplemented with 1% glycerol. When the crude extract was tested for antimicrobial activity, a clear bactericidal effect was observed on 79 strains (40%), a bacteriostatic effect on 25 strains (12%) and no effect in the case of 96 strains (48%). A very good inhibitory effect was noticed against numerous MRSA, MSSA, *Enterococci*, and *Enterobacteriaceae* isolates. For several environmental *E. coli* strains, the bactericidal effect was encountered at a violacein concentration below of what was previously reported. A different effect (bacteriostatic vs. bactericidal) was observed in the case of *Enterobacteriaceae* isolated from raw vs. treated wastewater, suggesting that the wastewater treatment process may influence the susceptibility of MDR bacteria to violacein containing bacterial extracts.

## Introduction

Since their discovery (e.g., penicillin in 1928), antibiotics have changed the face of medicine, saving millions of lives from deadly infections. But, in the recent context of a “post-antibiotic era”, the society is threatened by the spread of antimicrobial resistance (AMR) phenomenon and pan-resistant bacteria, i.e. deadly pathogens that do not respond to the treatment with existing antimicrobials. Thus, mechanisms to develop new and/or improved strategies that would reduce this issue need to be put into place. Warnings in this matter have been frequent and consistent, one of the latest reports^1^ stating clearly that if the AMR problem is not approached properly and no new antibiotics are developed, AMR will be the main cause of mortality by 2050, with more than 10 million deaths each year worldwide, surpassing even cancer.

Even though the discovery of antimicrobials has long been regarded as one of the most significant medical achievements of the past century, there was a constant decrease in the pharmaceutical industry’s interest for the development of new antibiotics. For example, the number of new antimicrobials approved for human use has decreased eightfold from mid-1980s (16 drugs) to 2010 (2 drugs)^2^. All this while the proportions of multidrug-resistant (MDR) pathogens such as methicillin-resistant *Staphylococcus aureus* (MRSA), vancomycin-resistant *Enterococcus* (VRE) and fluoroquinolone-resistant *Pseudomonas aeruginosa* (FQRP) have increased from below 5% in the 1980s to above 30% (VRE and FQRP) or even 60% (MRSA) in the 2000s^3^.

The downfall of antimicrobial drug discovery in the genomic era has revived the interest in screening natural products^4^. Historically, most antibiotic drugs were derived from natural environments and this trend continues in the recent period, as 50% of antibacterial compounds undergoing clinical trials in 2011 were of this nature^5^. Natural compounds and habitats are an important source of novel antibiotics in modern medicine, and have had an unquestionable impact on global health so far^6,7^. Among the extreme environments, Antarctica has proven to be a rich source of novel microbial species with increased potential for various applications, including the discovery of antimicrobial compounds^8–10^. The approach to use microorganisms specific to Antarctic habitats has proven successful, over 200 patents being filed related to biotechnological potential of these microbial resources^11^.

*Janthinobacterium* is a genus of Gram-negative bacteria that has been found in various environments, including Antarctica^12,13^. Some members of this genus are violet colored, a trait observed in other bacterial genera as well (e.g., *Chromobacterium*, *Collimonas*, *Duganella*, *Pseudoalteromonas* etc.)^14^. The violet color is given by violacein, a compound that has diverse biological activities, including a strong antibacterial effect against a wide array of important human pathogens^13,15–22^. However, the majority of MDR strains tested in these studies were clinical isolates, with a special attention given to MRSA, thus limiting the perspectives for application of violacein/bacterial extracts with violacein. According to the One Health approach^23^, it is necessary to expand the research focus and apply a multidimensional strategy to reduce the spread and effects of AMR on human and animal health, on human waste management, on agricultural and food production and aquaculture etc.^24^. For example, wastewaters and wastewater treatment plants are seen as potential reservoirs of antibiotic resistant bacteria (ARBs) and resistance genes (ARGs)^25–27^, contributing to their release into surface waters, recreational and drinking waters, soil and food products etc. Thus, new wastewater treatment strategies need to be developed to counteract the spread of ARBs and ARGs, and simultaneously be environmental-friendly.

The purpose of the current work was to assess to what extent a violacein-containing bacterial extract can be used to target MDR bacteria not only of clinical, but also environmental origin. For this, specific objectives were set: (i) to characterize a new variant of *J. lividum*, isolated from Antarctica and investigate its phenotypic and phylogenetic traits, together with the violacein yield; (ii) to test the violacein-containing extract against 200 MDR bacteria isolated from clinical and raw/treated wastewater samples, with different AMR patterns; (iii) to discuss the strain’s biotechnological potential in designing new strategies to reduce the burden of antibiotic resistance in a One Health approach, aimed at improving both human and environmental health.

## Results and Discussion

### ROICE173 phylogenetic analysis and phenotypic traits

**Figure 1:**
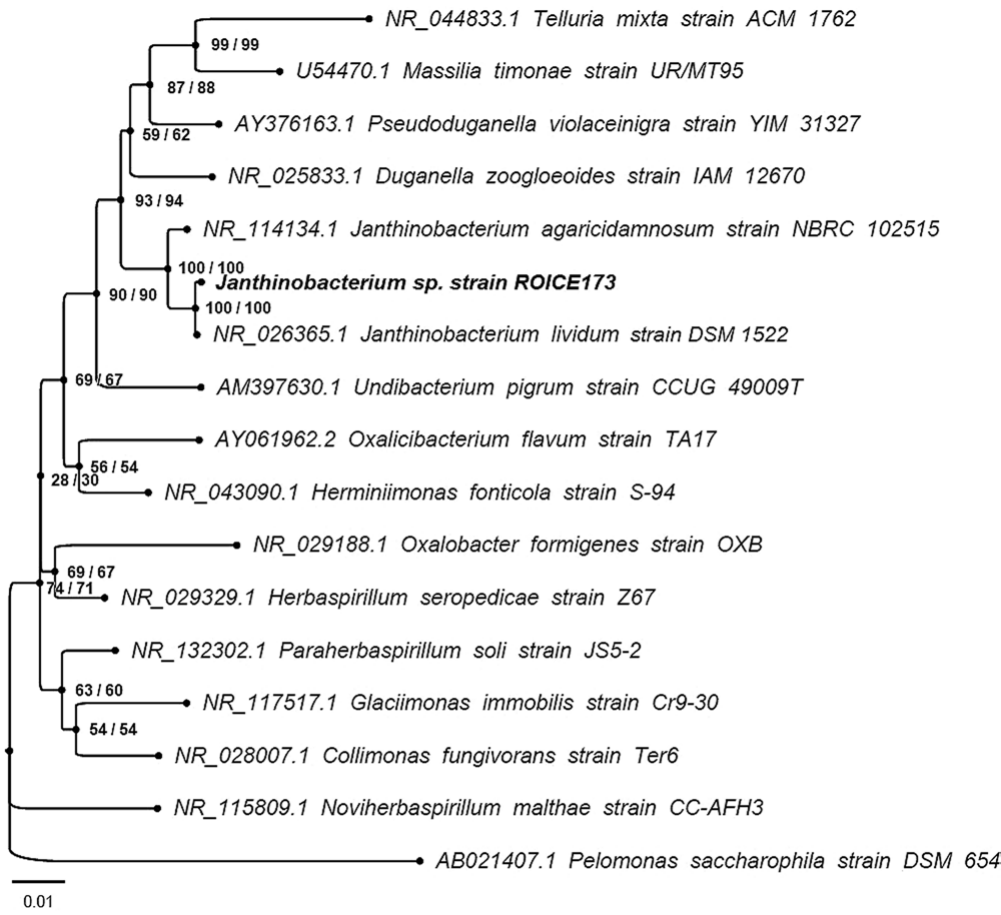
Maximum likelihood phylogenetic tree based on 16S rRNA gene sequences showing the position of strain ROICE173 among the related type species of the *Oxalobacteraceae* family. The numbers at nodes represent the bootstrap percentages derived from 1000 replications (maximum-likelihood/neighbour-joining methods). *Pelomonas saccharophila* strain DSM 654 was used as outgroup.

ROICE173 is a Gram-negative, pigmented bacterial strain isolated from snow samples collected from King George Island in West Antarctica during the Romanian ROICE expeditions. The comparative DNA sequence analysis revealed that ROICE173 is affiliated to the Gram-negative *Janthinobacterium* genus (Fig. 1). The sequence dissimilarity between the *J. lividum* type strain DSM 1522 ^T^ and ROICE173 is only 0.147%, whereas the same value is ~1% between *J. lividum* strain DSM 1522 ^T^ and *J. agaricidamnosum* strain NBRC 102515 ^T^, an observation previously made by Lincoln *et al*.^28^. Therefore, ROICE173 is most probably a new variant of the *J. lividum* species. Additionally, the stability of the nodes corresponding to the *Janthinobacterium* genus (100% bootstrap values) strongly supports this conclusion.

When grown on R2 broth, the shortest doubling time (hours) was observed at 25 °C (1.15), followed by room temperature (1.35), 20 °C (3.07), 15 °C (8.34) and 10 °C (8.47). On Nutrient broth, the following values were obtained: 25 °C (3.85), followed by room temperature (4.13), 20 °C (4.3), 15 °C (6.77) and 10 °C (6.93). No growth was observed at ≤5 °C or >30 °C, in neither of the two media tested.

From the 192 carbon sources tested using the PM1 and PM2 BIOLOG microplates, ROICE173 could metabolise 74 (Supplementary Table 2), the metabolic profile of carbohydrate degradation being similar to that of the *J. lividum* type strain^29^. However, several differences were found for ROICE173 when compared to the type strain, being observed for the first time an ability to metabolise several nucleosides (2-Deoxy Adenosine, Adenosine, Thymidine); amino acids (L-Histidine, L-Glutamine, Glycyl-L-Proline, D-Glucosamine, N-Acetyl-D-Glucosamine); acids (Acetoacetic acid, Citric Acid, m-Hydroxy Phenyl Acetic Acid, D-Malic Acid, L-Malic Acid, Pyruvic Acid, Butyric Acid, Fumaric Acid, Acetoacetic acid, D-Tartaric Acid); alcohols (L-Arabitol, Adonitol, m-Inositol); keto acids (5-Keto-D-Gluconic Acid); saccharides (L-Lyxose, D-Cellobiose, Maltotriose, Laminarin, Dextrin).

Regarding the osmotic/ionic response and pH, ROICE173 can be characterized as extremotolerant. The strain can grow at a concentration of 3% NaCl or higher (6% NaCl, in the presence of glutathione or trigonelline as osmoprotectants); the pH values ranged between 5.5–9.5, ROICE173 being able to metabolize various substrates at pH 9.5 (Supplementary Table 2). According to Gillis and Logan^29^, other typical or atypical *J. lividum* strains have an optimum pH of 7–8 and cannot grow above 2% NaCl. *J. lividum* ROICE173 can also sustain 5% Na_2_SO_4_, 20% Ethylene glycol, 2% HCOONa, 3% Urea, 2% C_3_H_5_NaO_3_, 100 mM Na_3_PO_4_, 100 mM (NH_4_)_2_SO_4_, 100 mM NaNO_3_ and 20 mM NaNO_2_.

ROICE173 was observed to be resistant to 98% of the antibiotics tested using the PM11 and PM12 microplates (Supplementary Table 2). Even though Antarctica is considered one of the least exposed environments to anthropogenic impact and other human-related activities, antibiotic resistance among bacterial isolates is widespread^30,31^. This phenomenon may be a consequence of the presence of antibiotics in the environment synthesized naturally or under a competitive stress caused by the harsh conditions and limited nutrients^32,33^. However, when compared to the antimicrobial resistance profile of the type strain, *J. lividum* DSM1522, it was observed that they share an almost identical pattern (Supplementary Table 2), suggesting a putative intrinsic and extended resistance to various drugs, irrespective of the environment from which *J. lividum* strains are isolated. These findings are in agreement with other studies showing a natural antibiotic resistance, especially in Gram-negative bacteria^34^.

### Pigment production

Even though the optimal growth conditions were tested on both R2 and Nutrient broths, spectrophotometric measurements at 575 nm showed a higher pigment production on R2 broth, between 20 and 30 °C (data not shown). Thus, the quantification of violacein production was investigated and described only for these conditions. The four temperatures selected for quantification of pigment production were 20 °C, 22 °C–regarded as “room temperature”, 25 °C and 30 °C, on R2 broth supplemented with 1% glycerol (v/v). Glycerol was shown to enhance the production of the purple pigment, as opposed to glucose, a violacein production inhibitor^35^. The violacein concentrations were expressed in µg·mL^−1^ of bacterial culture and mg·g^−1^ biomass as described in detail in the Methods section.

**Figure 2:**
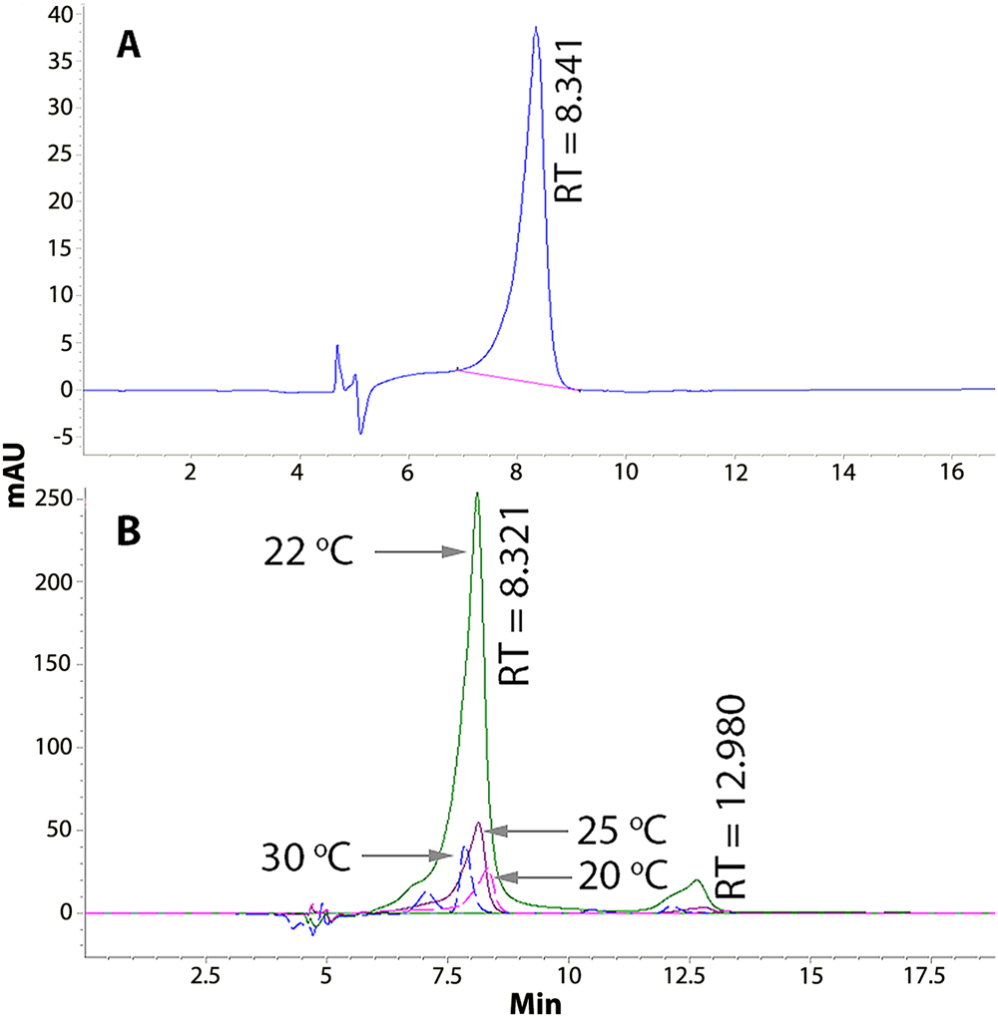
High-Performance Liquid Chromatography with Diode-Array Detection (HPLC-DAD) separation and retention times (RT) of pure violacein (**A**) and the *J. lividum* ROICE173 extracts at different temperatures.

**Figure 3:**
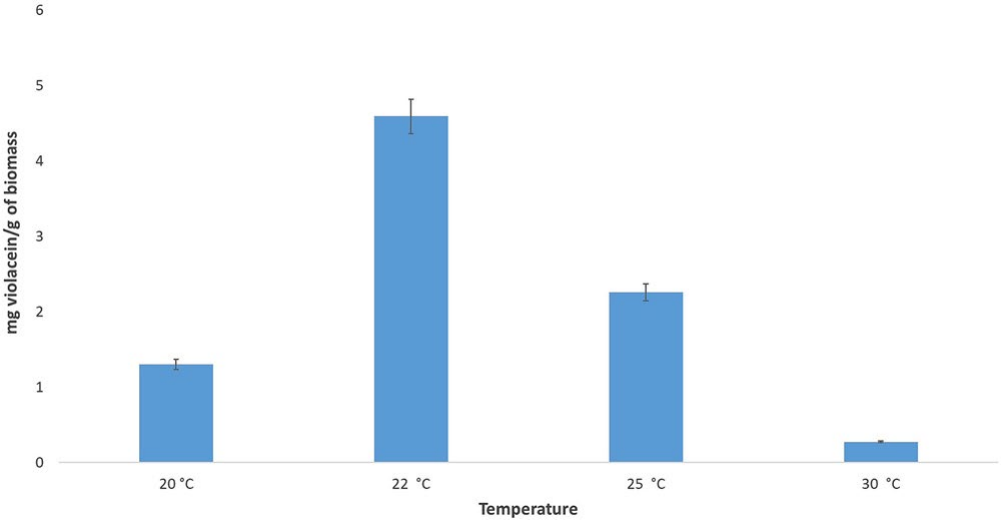
Violacein production by *J. lividum* strains ROICE173 after 24 h growth on R2 broth supplemented with 1% glycerol (v/v) and 150 rpm.

The standard violacein had a retention time (RT) of 8.341 min, as shown in Fig. 2A. The limits of detection and quantification were 1.28 µg·mL^−1^ and 4.27 µg·mL^−1^, respectively. The ROICE173 extracts from different temperatures all had a major peak around RT = 8.321 min (Fig. 2B), but also additional peaks at RT = 12.98 min and RT = 7.066 min (the 30 °C sample), that can be attributed to deoxyviolacein and oxyviolacein, respectively^36^. The violacein concentrations, expressed in µg·mL^−1^ of bacterial culture (and mg·g^−1^ biomass) determined for *J. lividum* ROICE173 ranged between 0.01 ± 0.0006 (0.27 ± 0.04) at 30 °C and 0.13 ± 0.028 (4.59 ± 0.78) at 22 °C, this being the highest concentration observed (Fig. 3). The highest violacein:deoxyviolacein ratio (10:1) was also observed at room temperature (data not shown). The results are comparable or even higher to violacein yields reported in other studies, e.g. a similar violacein concentration was observed for *J. lividum* from a glacier habitat only after more than 72 h incubation time^37^. However, for upscaling to bioreactor and/or (semi-) industrial applications, future studies have to be implemented to improve the violacein yield in ROICE173 as this process shows a high degree of variability, depending on contact with a substrate^13^, on the pH of the medium^38^, addition of carbon source^35^, shaking^39^, etc.

### Antimicrobial effect of ROICE173 extract against MDR bacteria

**Figure 4:**
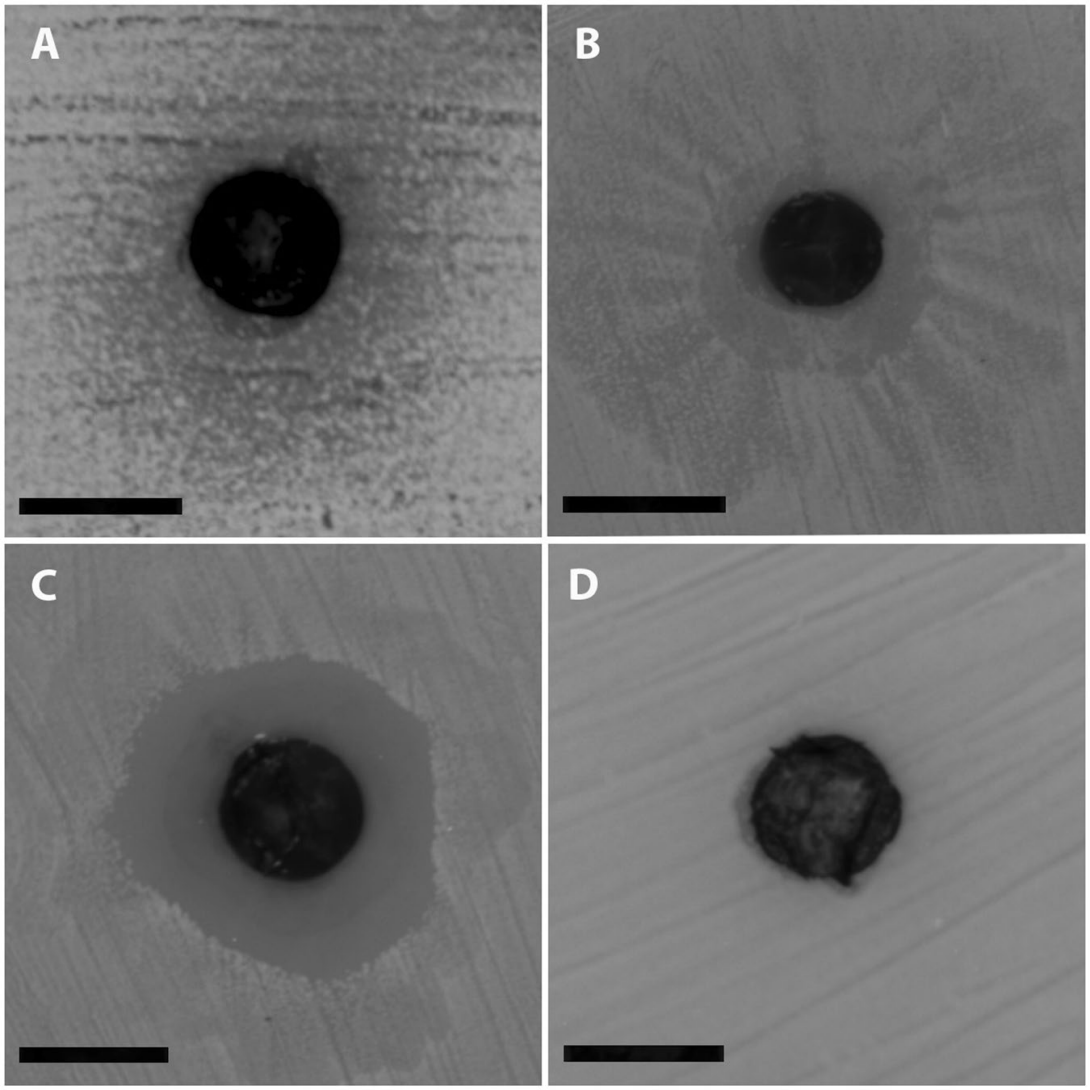
Types of effects of ROICE173 bacterial extract against the investigated MDR bacteria: (**A**,**B**) inhibition (bacteriostatic); (**C**) bactericidal; (**D**) no effect. Bar = 6 mm.

Overall, out of the 200 MDR bacteria tested, the crude extract corresponding to 30 µg violacein in a ratio of 10:1 with deoxyviolacein, showed an inhibitory (bacteriostatic) effect (Fig. 4A,B) against 25 strains (12%), a clear bactericidal effect (Fig. 4C) against 79 strains (40%), and no effect (Fig. 4D) against 96 strains (48%). Detailed information on the bacteriostatic/bactericidal effect is presented in Supplementary Table 3.

In the attempts to set the epidemiological cut-off values (CO_WT_) for inhibition zone diameters, the mean minus 2.5 standard deviation (SD) was used, thus including 99.4 of the wild-type (WT) observations. According to the normalized resistance interpretation (NRI) statistical method, they were ≥7 mm for *Janthinobacterium lividum* ROICE173 extract against *Enterobacteriaceae,* ≥8 mm against *Enterococcus* spp. and ≥10 mm on *Staphylococcus* sp. (Table 1). Epidemiological cut-off values based on *in vitro* susceptibility data are a significant phenotypic system providing information on the trend of microbiological resistance, having no inherent clinical significance.

**Table 1:** Epidemiological cut-off values (CO_WT_) calculated by NRI analysis of inhibition zone data with *Janthinobacterium lividum* ROICE173 extract.

Bacterial taxa	Mean inhibition zone (mm)	SD	CO_WT_ (mm)
*Enterobacteriaceae* (environmental)	11.98	1.87	≥7
*Enterococcus* spp. (environmental)	12	1.48	≥8
*Enterococcus* spp. (clinical)	11.85	1.46	≥8
*Staphylococcus* spp. (clinical)	16.79	2.53	≥10

When the ROICE173 extract was tested against environmental strains, the most frequent size of inhibition zone was between 11 to 13 mm, coinciding with the average values calculated by NRI analysis (11.98 mm for *Enterobacteriaceae* and 12 mm for *Enterococcus* spp.) (Table 1). The sizes of inhibition zones in relation to resistance were balanced, as it can be seen in histograms (Supplementary Fig. 1A,B). For the clinical isolates, the average inhibition zones calculated by NRI analysis were 11.85 mm in case of *enterococci* and 16.79 mm for staphylococci (Table 1). Clinical isolates were not homogenous in antibiotic resistance, and a balance between the wild-type (WT) and nonwild-type (NWT) strains was not observed (Supplementary Fig. 1C,D).

### Effect of *J. lividum* ROICE173 extract on MDR *enterococci*

*Enterococci* are Gram-positive bacteria that colonize the natural intestinal flora of humans and animals^40^ and have been traditionally used as indicators of faecal pollution of different water bodies. Particularly, *E. faecalis* and *E. faecium* are the two species most frequently associated with a range of enterococcal diseases in clinical settings (urinary tract infections, peritonitis, endocarditis etc.), being responsible for one-third of all nosocomial infections worldwide^41^. Vancomycin-resistant *Enterococci* (VRE) have become a serious health problem worldwide, being encountered in Europe, USA, and Asia, in recreational waters, in surface and drinking water^42–44^. Recently, VRE *E. faecium* has been included in the “High” category of the World Health Organization (WHO) priority pathogens list for research and development of new antimicrobials^45^. Another important issue related to VRE is that the *van* resistance genes can be transferred *in vivo* from *E. faecalis* to multiresistant *S. aureus*^46^, seriously compromising the usefulness of vancomycin. Despite these issues, very few studies have investigated the effect of bacterial extracts with violacein on these bacteria.

**Figure 5:**
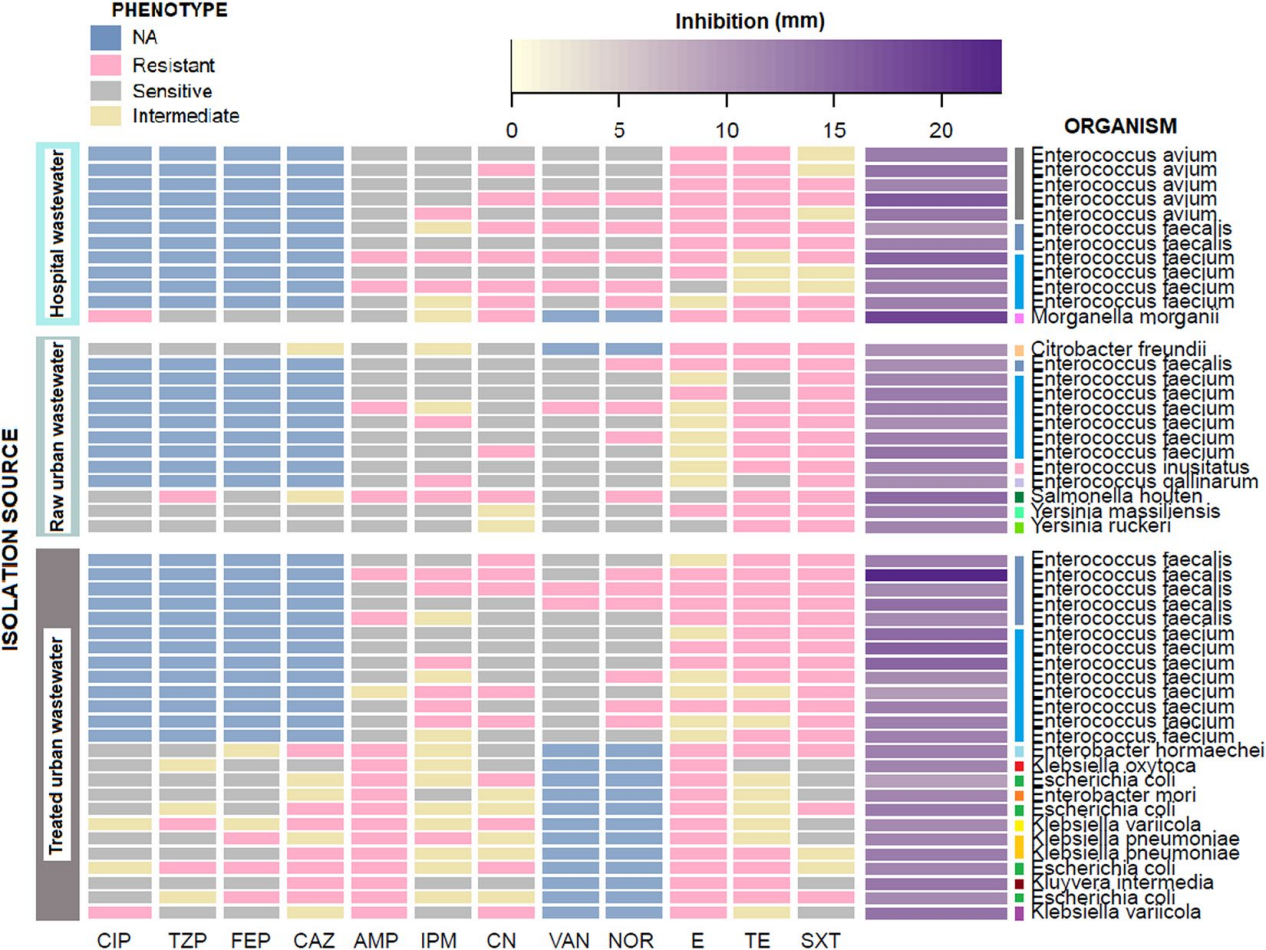
Heatmap of the bactericidal effect (mm) of *J. lividum* ROICE173 crude extract against environmental MDR bacterial isolates. CIP = Ciprofloxacin; TZP = Tazobactam; FEP = Cefepime; CAZ = Ceftazidime; AMP = Ampicillin; IPM = Imipenem; CN = Gentamicin; VAN = Vancomycin; NOR = Norfloxacin; E = Erythromycin; TE = Tetracycline; SXT = Trimethoprim-sulfamethoxazole.

**Figure 6:**
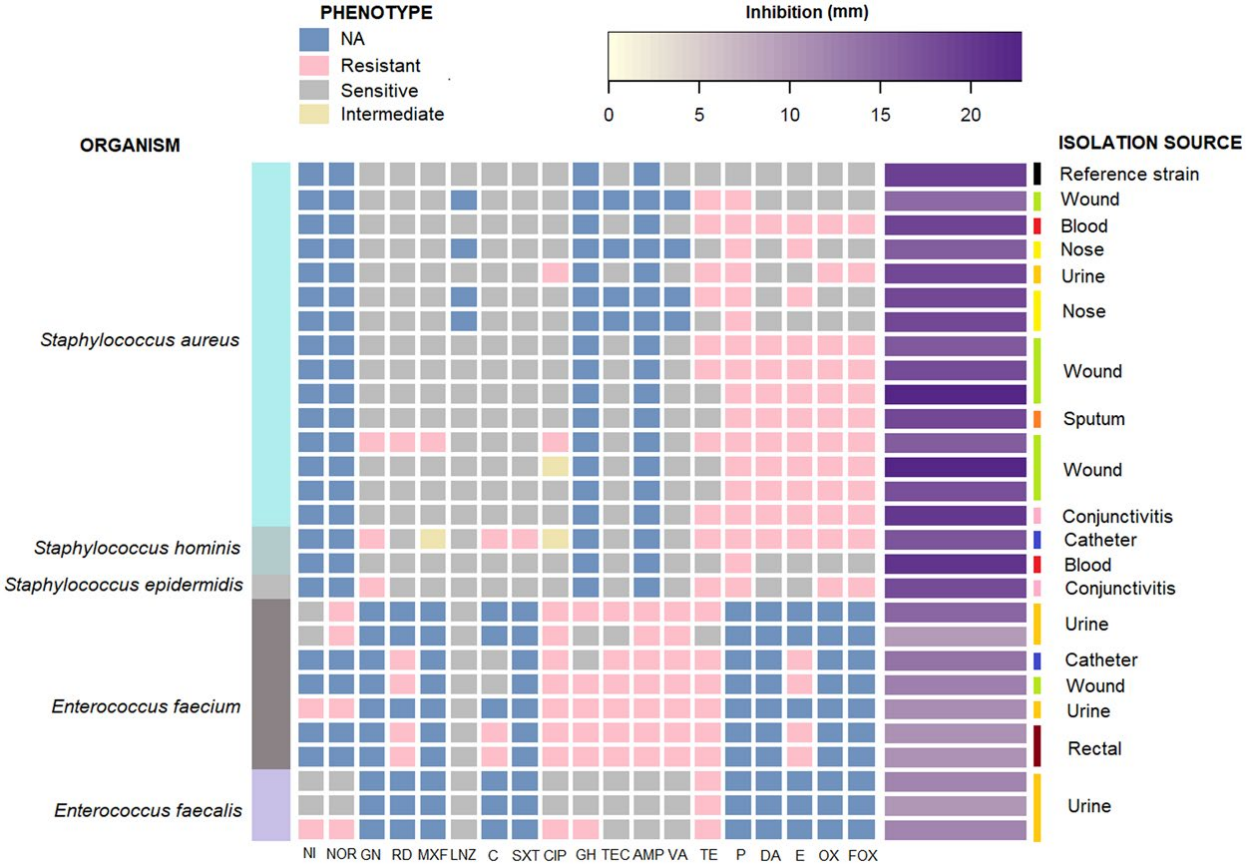
Heatmap of the bactericidal effect (mm) of *J. lividum* ROICE173 crude extract against clinical MDR bacterial isolates. NI = Nitrofurantoin; NOR = Norfloxacin; GN = Gentamicin; RD = Rifampin; MXF = Moxifloxacin; LNZ = Linezolid; C = Chloramphenicol; SXT = Trimethoprim-sulfamethoxazole; CIP = Ciprofloxacin; GH = Gentamicin high; TEC = Teicoplanin; AMP = Ampicillin; VA = Vancomycin; TE = Tetracycline; P = Penicillin; DA = Clindamycin; E = Erythromycin; OX = Oxacillin; FOX = Cefoxitin.

The ROICE173 extract showed a bactericidal effect against various MDR *enterococci*, regardless of the isolation source (Supplementary Table 3, Figs 5 and 6). Out of the 43 *Enterococcus* sp. strains where a clear antimicrobial effect was observed (several examples presented in Supplementary Fig. 2), 14 were VRE *E. avium*, *E. fecalis*, *E. faecium*. The extract showed a better antimicrobial activity on vancomycin-resistant *E. faecium* and *E. fecalis*, with inhibition zones >15 mm, than against *E. avium*, where the inhibition response was smaller (Fig. 5). Previous investigations have shown either a low effect of violacein alone against *Enterococci*^47^ or an inhibitory effect only at concentrations >68 µg·mL^−1^ ^15^. Thus, the results of the present study suggest that the violacein synthesized by ROICE173 or the presence of deoxyviolacein together with violacein, in the ratio 1:10, may enhance the antimicrobial activity. However, future investigations have to be undertaken to observe the structural diversity of violacein synthesised by ROICE173 or the violacein-deoxyviolacein synergy.

### Effect of *J. lividum* ROICE173 extract on MDR *Enterobacteriaceae*

The *Enterobacteriaceae* are a very important group of bacteria in medical microbiology. It includes facultative and obligate pathogens and is considered a reservoir of resistance genes and a frequent contaminant of food and water. For example, Frigon *et al*.^48^ have observed that the level of potentially pathogenic *E. coli* in natural waters is comparable to that in wastewater samples, with an abundance of 29–32% and 14–31%, respectively.

The ROICE173 extract was tested against a wide array of MDR *Enterobacteriaceae* (Supplementary Table 1), revealing a bactericidal or bacteriostatic effect against several medically important groups, such as: *Citrobacter*, *Escherichia coli*, *Klebsiella*, *Morganella*, *Salmonella*, *Yersinia* (Supplementary Table 3, Fig. 5), at lower violacein concentration than previously reported^16,21^.

No effect was observed when the bacterial extract was tested on *Enterobacteriaceae* and *E. coli* clinical isolates. This is in agreement with previous studies that have shown either a negative/poor effect on Gram-negative strains, including clinical *E. coli*^15,36^ or an inhibitory effect only at concentrations of 68 µg·mL^−1^ or higher^16,21^. In the *Enterobacteriaceae* strains isolated from raw wastewater, the effect of ROICE173 extract was mostly bacteriostatic for *Citrobacter*, *Cronobacter*, *Enterobacter*, *E. coli*, *Klebsiella* and *Rahnella* (Supplementary Table 3). A clear inhibition zone was observed only for some *Citrobacter*, *Salmonella*, and *Yersinia* strains (Fig. 5), in the range of 10 ± 1–14 ± 2 mm. However, when the same extract was applied for the treated wastewater isolates, a clear bactericidal effect could be observed, regardless of the taxonomical affiliation (Supplementary Table 3, Fig. 5). Here, the inhibition areas varied between 9 ± 1 and 13 ± 2 mm. This observation has raised the hypothesis that the wastewater treatment process may influence the AMR pattern of selected bacteria, in some cases making them more susceptible to antimicrobial compounds such as violacein. The majority of studies have found an increase in the prevalence of antimicrobial resistance and/or ARGs in microbial populations associated with wastewater treatment plants^49,50^. However, other studies have shown that the microbiological quality of the treated effluent is strongly influenced by the treatment efficiency^51^ or that the physicochemical process of wastewater treatment reduces not only the abundance of resistant *E. coli*, but also the average frequency of multiple ARG classes in the ARG-carrying *E. coli* isolates^52^. This could lead to an increase in antimicrobial sensitivity. Other previous studies investigating the antibiotic resistance in *Enterobacteriaceae* isolated from the wastewater influent and treated effluent found a decrease in coliform counts but no significant changes in AMR and MDR bacteria, the high similarity being found regarding the AMR phenotypes frequency and diversity before and after wastewater treatment^53^. However, the issue of increased susceptibility to violacein of bacteria from wastewater treatment plant effluent needs further investigations to supplement the *E. coli* and *Enterobacteriaceae* strains presented in this study, but for which data regarding the impact of wastewater treatment on the prevalence of antimicrobial resistance is missing.

### Effect of *J. lividum* ROICE173 extract on MDR Staphylococci

Staphylococci are bacterial pathogens associated with a wide range of human infections, including skin infections, pneumonia, and septicemia^54^. Infections with this microorganism can be difficult to treat because their antibiotic resistance has increased over time, MRSA posing serious health problems since 1961^55^. Community acquired MRSA infections are more frequent, this important MDR pathogen being encountered in various environments outside the clinical area^56,57^.

The *Staphylococcus aureus* strains used for testing in this study were isolated from blood, wound, urine, sputum, conjunctival and nasal samples. Five strains were MSSA (Methicillin-sensitive *Staphylococcus aureus*) and ten were MRSA. 90% of the MRSA strains were resistant to erythromycin, 60% to tetracycline, 20% to ciprofloxacin and 10% to gentamicin. None of the MRSA strains was resistant to glycopeptides and oxazolidinones. Besides the *S. aureus* strains, the ROICE173 extract was tested also on coagulase-negative staphylococci - two *Staphylococcus hominis* and one *Staphylococcus epidermidis* strains, isolated from blood, catheter, and conjunctival samples, as these coagulase-negative staphylococci are frequently associated with nosocomial infections, many being line infections.

The ROICE173 crude extract showed a bactericidal effect against all the *Staphylococcus* strains tested, with inhibition zones between 14 ± 1 and 22 ± 2 mm (Fig. 6). Similar inhibition areas were also identified by Wanga *et al*.^36^, who observed also that violacein seemed to be more specific against *S. aureus* at lower concentrations. Our results, using crude extract, are in line with numerous other studies that have investigated the antimicrobial effect of violacein from different compound producing strains (*Chromobacterium violaceum*, *Duganella* sp., *Janthinobacterium lividum*; *Collimonas fungivorans* etc.) on different *S. aureus* and *S. epidermidis* strains. The strains tested in previous investigations were similar in isolation sources to those tested in this study: human carbuncle^58^; tissue, blood, sinus, sputum or bone^18^; clinical isolates^20,21^, etc.

### Ecotoxicological effect investigation of ROICE173 extract on *Artemia franciscana* nauplii

Aquatic invertebrates are widely used in ecotoxicological studies, because they are relatively easy to maintain under test conditions, their use does not imply ethical concerns, and they are generally more sensitive than vertebrates or plants to a various range of pollutants^59,60^.

The extract from *J. lividum* ROICE173 strain was tested in ten increasing concentrations on *Artemia franciscana* nauplii, in the range of 30 µg·mL^−1^ of violacein (1X bactericidal concentration) and 900 µg·mL^−1^ of violacein (30X bactericidal concentration). As the death rate of the controls was <10%, the tests were considered relevant, according to the manufacturer’s instructions. The concentration ≥780 µg·mL^−1^ of violacein (26X) in the extract led to the death of all the *A. franciscana* larvae (data not shown). The mortality rates of *A. franciscana* after 24 h when exposed to 30–720 µg·mL^−1^ of violacein (1X–24X bactericidal concentration) are shown in Supplementary Fig. 3. The LC_50_ value varied between 649.17–659.81 µg·mL^−1^ of violacein. Previous investigations were performed recently by Neu *et al*.^61^, but only for 4.25 and 0.42 µg·mL^−1^ of violacein, stating that no significant effect on the survival of *Artemia* nauplii was observed.

### Perspectives and applications

*Janthinobacterium lividum* ROICE173 strain is a promising candidate towards the development of new antimicrobials with clinical relevance, as violacein has a valuable antimicrobial activity observed either in this study or in other experiments^16^. Also, it was observed that violacein has a synergistic potential with several antibiotics (e.g., gentamicin, cefadroxil, azithromycin, kanamycin etc.)^20,58^. Violacein–gentamicin and violacein–cefadroxil combinations showed low minimum inhibitory concentrations (MIC) of 1.0 μg·mL^−1^ against *Staphylococcus aureus*. Violacein–macrolide and violacein–aminoglycoside, together with violacein–azithromycin and violacein–kanamycin class combinations revealed exhibited medium or significant synergy against *Salmonella typhi*. Also, in another study^15^, Martins *et al*. have shown that nanoparticles loaded with violacein were two to five times more effective than violacein alone against MRSA, justifying its possible real application in medical industries. Violacein also has an anti-proliferative activity against cancer cells lines^62^, anticancer activity against human breast cancer cells in a time and dose-dependent manner^63^ and antifungal activity^64^.

As mentioned in the introduction, the environment plays an important role in the dissemination of ARBs and ARGs, as they are considered new classes of pollutants^65,66^. These contaminants were detected in various aquatic habitats (e.g. wastewaters, surface and groundwater, drinking water) and in the soil and sediments^27,67^. The basic concept of the One Health approach is that the AMR problem has to be tackled on multiple levels and not just the clinical. Thus, in this context, the use of *Janthinobacterium lividum* ROICE173 in designing biotechnological applications to reduce the burden of environmental AMR via new or improved wastewater treatment technologies can be proposed. The crude extract had a bactericidal effect against various MDR microorganisms from several genera (*Enterococcus* and *Morganella* in hospital wastewaters; *Enterococcus*, *Salmonella*, and *Yersinia* in raw urban wastewaters; *Enterococcus*, *Enterobacter*, *E. coli*, *Klebsiella* etc. in wastewater treatment plant effluent). The strain’s physiological traits, allowing its survival in high salinity and high pH conditions, corroborated with its ability to metabolize different carbon sources, represent important features to take into consideration in such an approach. Also, the fact that ROICE173 is resistant to numerous antibiotics, which seems to be an intrinsic characteristic of the *Janthinobacterium* genus, gives this strain a competitive advantage in polluted environments such as wastewaters, as it is well known that these habitats contain high levels of antibiotic residues^27^. Also, the release of violacein from the bacteria into the environment seems to have a limited ecotoxicological effect, as it was observed during the tests performed on *Artemia* larvae, with an LC_50_ that is 20-fold higher than the bactericidal concentration.

However, several aspects have to be taken into consideration in future studies when proposing these new approaches in healthcare and environmental biotechnology. Even though a single case of septicaemia related to an infection with *Janthinobacterium lividum* was reported^68^, this issue requires extended research, as genomic analysis of another violacein-producing bacteria (*Chromobacterium violaceum*) led to the identification of many putative virulence factors, none of which have been characterized before^69^. Recently, violacein from *Chromobacterium violaceum* was observed to be toxic to human embryonic kidney (HEK293) and human fetal lung fibroblast (IMR90) cell lines^70^. Thus, further toxicity tests on different human cell lines and other model eukaryotic organisms (e.g., the microalga *Selenastrum capricornutum* and *Daphnia magna*) need to be performed.

In conclusion, the current study was focused on the ROICE173 bacterial strain isolated from Antarctic snow samples. Based on the phylogenetic analysis, the strain belongs to the *Janthinobacterium lividum* species. The optimum doubling time was observed on R2 broth, at 25 °C. However, the best violacein yield of 4.59 ± 0.78 mg·g^−1^ biomass was obtained at 22 °C, on R2 broth supplemented with 1% glycerol (v/v). The ROICE173 crude extract, corresponding to 30 µg of violacein and a ratio violacein:deoxyviolacein of 10:1, was tested against 200 MDR bacteria isolated from clinical and environmental samples, with different resistance patterns to aminoglycosides, carbapenems, cephalosporins, fluorquinolones, glycopeptides, macrolides, penicillins, tetracyclines, aztreonam, trimethoprim-sulfamethoxazole, rifamycin- rifampicin, oxazolidone-linezolide, cloramphenicol, polimixins- colistin, nitrofurantoin. The extract showed a clear bactericidal effect against 79 strains (40%), a bacteriostatic effect against 25 strains (12%) and no effect against 96 strains (48%). A very good inhibitory effect was observed against both environmental and clinical isolates such as MRSA and MSSA, *Enterococci*, and *Enterobacteriaceae*. For some species (e.g., environmental *E. coli*), the bactericidal effect was observed at a violacein concentration below of what was previously described. However, this may be related to the synergistic effect with deoxyviolacein and requires future investigations. A different effect (bacteriostatic vs. bactericidal) was observed on *Enterobacteriaceae* strains isolated from raw vs. treated wastewater, suggesting that the wastewater treatment process may influence the susceptibility of MDR bacteria to violacein containing bacterial extracts. Overall, *J. lividum* ROICE173 is a promising candidate for biotechnological applications aimed at developing new antimicrobials or at reducing the spread of MDR bacteria into the environment. Thus, it may contribute to the efforts of diminishing the antimicrobial resistance phenomenon in a One Health approach, targeting human, animal and environmental health altogether.

## Methods

### Isolation of *J. lividum* ROICE173 and growth conditions

*Janthinobacterium lividum* ROICE173 was isolated from snow samples collected from the King George Island in West Antarctica, during the ROICE Romanian expedition in 2015. The samples were thawed overnight and processed in the laboratory of KOPRI-King Sejong research station as follows: the melted snow was filtered onto 0.2 µm MCE membranes (Fioroni, France) and the membranes were kept in R2 broth (composition in g L^−1^: casein 0.25; peptone 0.25; casaminic acids 0.5; yeast extract 0.5; dextrose 0.5; starch 0.5; dipotassium phosphate 0.3; magnesium sulphate 7H_2_O 0.05; sodium pyruvate 0.3 g; pH 7 ± 0.2). The isolation of pure cultures was carried out in the Environmental Microbiology Laboratory at the Institute of Biological Research Cluj-Napoca by performing serial dilutions and inoculation on R2A medium (R2 broth supplemented with 15 g L^−1^ agar). The plates were incubated aerobically, at 10 °C, in a 352H-PE Climate Chamber (Sanyo). Single colonies were selected based on morphology and/or pigmentation, re-streaked several times until pure cultures were obtained. The target strain displayed a violet color and was selected for future investigations with the code ROICE173.

The optimal growth temperature for an enhanced yield of bacterial biomass was investigated on R2 and Nutrient broths: 2, 5, 10, 15, 20, 25, 30, 37 °C. The inoculum was prepared by transferring a single colony in 100 mL liquid medium in 250 mL sterile Erlenmeyer flasks and grown until optical density OD_600nm_ = 1. This step was used as starting point for the experiment, in which 100 mL medium was inoculated in 250 mL sterile Erlenmeyer flasks, in triplicate, at a starting OD_600nm_ = 0.05. The flasks were incubated at the selected temperature for 72 h in a 352H-PE Climate Chamber (Sanyo) and, at set times, aliquots were taken to determine the culture OD_600nm_ using a UV-1700 UV-VIS spectrophotometer (Shimadzu, Japan).

### Phenotype MicroArray Assay

Phenotype MicroArray (PM) plates (BIOLOG, Hayward, USA)^71^ were used to observe various phenotypic traits of ROICE173. The following PM plates were used: PM1, 2 - carbon utilization assays; PM9 - osmotic/ionic response; PM10 - pH response; PM11, 12 - chemical sensitivity to different antibiotics and xenobiotics. Preparation of the different IF (Inoculating Fluids; supplied by BIOLOG) solutions and inoculation of the PM plates was performed according to the BIOLOG PM protocol for *E. coli* and other Gram-negative bacteria with dye H. The strains were grown overnight on R2A at 25 °C and isolated colonies were removed from the agar plate using a sterile swab, added to a tube containing 16 mL of IF-0 solution and further used as working inoculums in preparing the inoculation solutions. Finally, 100 μL of the final cell suspension was inoculated to each well. Plates were incubated at 25 °C for 96 h in a BE-400 incubator (Memmert, Germany) and the phenotypic response was evaluated spectrophotometrically using a Synergy HTX microplate reader (BioTek, Germany), the values of OD590 minus OD750 being used to quantify the color changes.

### DNA isolation, 16S rRNA gene amplification, sequencing and phylogenetic analysis

A single colony of ROICE173 grown on R2A medium was used to inoculate 100 mL of R2 broth in a sterile 250 mL Erlenmeyer flask and grown overnight at 25 °C. The culture was centrifuged for 15 min at 5000 × g, the supernatant discarded and the pellet used for DNA extraction using the ZRSoil Microbe DNA kit (ZymoResearch, USA), according to the manufacturer’s instructions. The concentration and quality of the extracted DNA were determined with a NanoDrop spectrophotometer (Thermo Scientific, Wilmington, DE, USA) (data not shown). DNA samples were stored at −20 °C until further analysis. The 16S rRNA gene was amplified with specific bacterial primers 27FB (5′-AGAGTTTGATCCTGGCTCAG-3′) in combination with the universal primer 1492R (5′-ACGGHTACCTTGTTACGACTT-3′). The PCR assay was performed with T-Gradient Thermal Cycler (Biometra, Germany) using the following conditions: 95 °C for 3 min, followed by 35 cycles of 95 °C for 30 s, 55 °C for 30 s and 72 °C for 1 min and 30 s, with a final extension of 10 min at 72 °C. The purified PCR products were sequenced by Sanger method as an externalized service (Macrogen Europe, The Netherlands). The corresponding 16S rRNA gene sequence of strain ROICE173 was deposited in GenBank under the accession number MG930775.

Phylogenetic analysis of the retrieved 16S rRNA gene sequence for ROICE173 strain included the gene sequence of *J. lividum* strain DSM 1522 ^T^ and *J. agaricidamnosum* strain NBRC 102515 ^T^, as well as the bacterial type strains of the other genera affiliated to the *Oxalobacteraceae* family. The 16S rRNA gene sequence of *Pelomonas saccharophila* strain DSM 654 was used as an outgroup. The analysis employed the Neighbour-Joining (NJ) and Maximum Likelihood (ML) algorithms as implemented in MEGA7^72^. The best fit substitution model was determined in MEGA7. The confidence of the tree topology was established through bootstrap analysis, including 1000 resamplings of the analyzed gene sequences. The evolutionary distances in the NJ tree were computed using the Kimura 2-parameter^73^ and the rate of variation among sites was modeled with a gamma distribution (G parameter = 0.43). The ML phylogenetic tree was also generated using the Kimura 2-parameter model, using a discrete gamma distribution in order to take into consideration the evolutionary rate differences among sites (G parameter = 0.43). The rate variation model allowed for some sites to be evolutionarily invariable (65% of sites). The analysis involved 17 gene sequences and a total of 1354 positions.

### Quantification of violacein by High-Performance Liquid Chromatography with Diode-Array Detection (HPLC-DAD)

Single colonies were grown overnight in R2 broth at 25 °C, in darkness, in continuous shaking at 150 rpm. The culture was used to inoculate ten sterile 250 mL Erlenmeyer flasks, each containing 100 mL R2 broth supplemented with 1% glycerol (v/v)^74^. The flasks were incubated in triplicate at four different temperatures (20 °C, room temperature = 22 °C, 25 °C and 30 °C), in darkness, in continuous shaking at 150 rpm. After 24 h, the culture was collected by centrifugation for 20 min at 5000 × g and 4 °C. The supernatant was discarded. The pellet was weighed to determine the wet biomass (referenced hereafter as “mg of biomass”) and resuspended in a volume of ethanol corresponding to ten times the weight of biomass (i.e. 10 mL ethanol for each mg of biomass). The solution was homogenized by ultrasonication for 1 hour using a Labsonic ultrasonic homogenizer (Sartorius, Germany), in continuous agitation on a magnetic stirrer, followed by another step of centrifugation as described above. The crude extract was concentrated at 50 °C to a final volume of 5 mL.

The crude violacein sample was analyzed using a HPLC 1200 (Agilent, USA) with Diode-Array Detection, as described previously^75^, with slight modifications. For this, 20 μL samples of the ethanol extracted violacein were analyzed using a C-18 column (Phenomenex Luna 5 μ C18(2) 100 Å, 250 × 4.6 mm) at 30 °C. The mobile phase was denatured ethanol (HPLC Grade, Sigma-Aldrich, USA): water, (50:50, v/v) and detection was performed at 575 nm using a G 1315 D detector. The violacein concentration within the ethanol extracts was determined as described above using crude violacein (Biomol GmbH, Germany, Cat. No. BVT-0473-M005) as standard.

### Antimicrobial activity assay

The ROICE173 crude extract in ethanol obtained at room temperature was tested for antimicrobial activity by agar well diffusion method^76^. 30 µg of violacein were used per each well. the ratio of violacein:deoxyviolacein being 10:1. Absolute ethanol was used as negative control. The 200 MDR bacteria tested were isolated either from environmental samples (hospital and urban wastewaters) (150 strains deposited in the EnviroAMR culture collection from the Institute of Biological Research Cluj-Napoca) or from clinical samples (50 strains deposited in the culture collection from the “Dr. Victor Babeș” Infectious and Tropical Diseases Hospital, Romania) (Supplementary Table 1). The strains were distributed among different genera, being mostly *Enterococci*, *Enterobacteriaceae* and *Staphylococcus* with various antibiotic resistance patterns as described in Supplementary Table 1.

The strains were grown overnight on Tryptone Soy Agar plates at 30  °C and transferred to 0.85% NaCl sterile solution at a cell density corresponding to 0.5 McFarland standard. Sterile cotton swabs were used to inoculate 20 mL of Mueller Hinton Agar plates and 6 mm wells were bored in the agar using sterile cork borers. The plates were incubated for 1 h at 4 °C followed by 24 h at 35 °C and the zone of inhibition was measured in mm.

Regarding bio-containment issues, pathogen cultures were performed by trained personnel in a Class II microbiological safety cabinet equipped with UV-lamp. A working space that is separated from the routine work was used in order to limit the potential exposure to pathogens. Glassware and sharp objects were eliminated from the cabinet interior and surfaces were cleaned and sterilized before and after each working session. Disposable gloves were worn when handling pathogens and disposable, sterile, cell-spreaders were used during inoculation procedures. Biological waste was sterilized before discarding using heat-resistant, autoclavable polypropylene bags.

### Toxicity assay on *Artemia franciscana*

The Artoxkit M (MicroBioTests Inc., Belgium) was used to perform a 24 h LC50 bioassay in a multiwell test plate using instar II-III larvae of the brine shrimp *Artemia franciscana*, according to the manufacturer’s instructions. Briefly, the cyst hatching was initiated 30 h prior to the start of the toxicity test in standard seawater, at 25 °C and 5000 lux in a 352H-PE Climate Chamber (Sanyo). Three independent experiments were performed, each concentration in triplicate, in ten increments in the range of 30 µg·mL^−1^ of violacein (1X concentration) and 900 µg·mL^−1^of violacein (30X concentration). The plates were incubated for 24 h at 25 °C, in darkness.

### Data analysis

Each of the experiments was performed in triplicate for error analysis. The standard deviations are presented as error bars in each graph where appropriate.

The heatmap for antibiotic resistance and violacein sensitivity was obtained with the heatmap.2 function of the gplots package in R.

The normalized resistance interpretation (NRI) method was applied to antimicrobial activity results for violacein extract. The average, SD and epidemiological cut-off values (CO_WT_) were calculated for the inhibition zone data sets by application of NRI analysis^77,78^ for *Staphylococcus* sp., *Enterococcus* sp. and *Enterobacteriaeae*. The smooth of the distribution for the observed frequencies was performed according to the standardized protocol that uses 4-point rolling means. The NRI method was used with permission from the patent holder, Bioscand AB, TÄBY, Sweden (European Patent No. 1383913, US Patent No. 7,465,559). The automatic and manual excel programmes were made available through courtesy P. Smith, W. Finnegan, and G. Kronvall.

Probit regression analysis was applied for the determination of LC_50_ of *Artemia franciscana* nauplii, using the XLSTAT package (Microsoft Excel).

## Electronic supplementary material

Supplementary figures

Supplementary Table 1

Supplementary Table 2

Supplementary Table 3

## Data Availability

All data generated or analysed during this study are included in this published article (and its Supplementary Information files). The 16S rRNA gene sequence will be made public by NCBI-GenBank on February 17^th^. If needed, the sequence can be provided by the corresponding author on request.
