# Supplementary material for: Investigating the potential use of an Antarctic variant of Janthinobacterium lividum for tackling antimicrobial resistance in a One Health approach
Source: Sci Rep. 2018 Oct 15;8:15272. doi: 10.1038/s41598-018-33691-6 (PMC6189184; doi:10.1038/s41598-018-33691-6)
Supplement: Supplementary file 1 — Supplementary figures [file 41598_2018_33691_MOESM1_ESM.pdf]

SUPPLEMENTARY FIGURES

Investigating the potential use of an Antarctic variant of *Janthinobacterium lividum* for tackling antimicrobial resistance in a One Health approach

Andreea Baricz, Adela Teban, Cecilia Maria Chiriac, Edina Szekeres, Anca Farkas, Maria Nica, Amalia Dascălu, Corina Oprișan, Paris Lavin, Cristian Coman

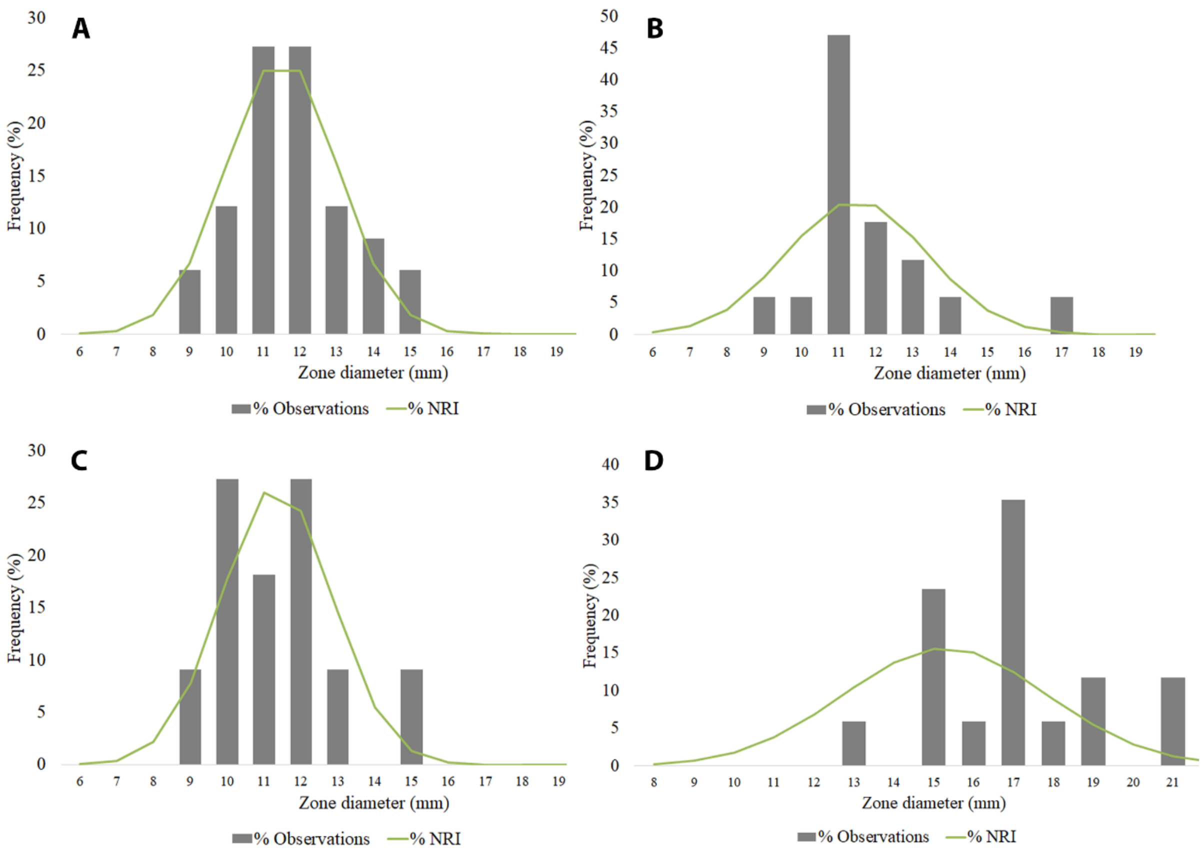

Suppl. Fig. 1. Frequencies of the zone diameters and normalized resistance interpretation for *Janthinobacterium lividum* ROICE173 extract with bactericidal effect against MDR bacteria. **A** - Environmental *Enterococcus*. **B** - Environmental *Enterobacteriaceae*. **C** - Clinical *Enterococcus*. **D** - *Staphylococcus*.

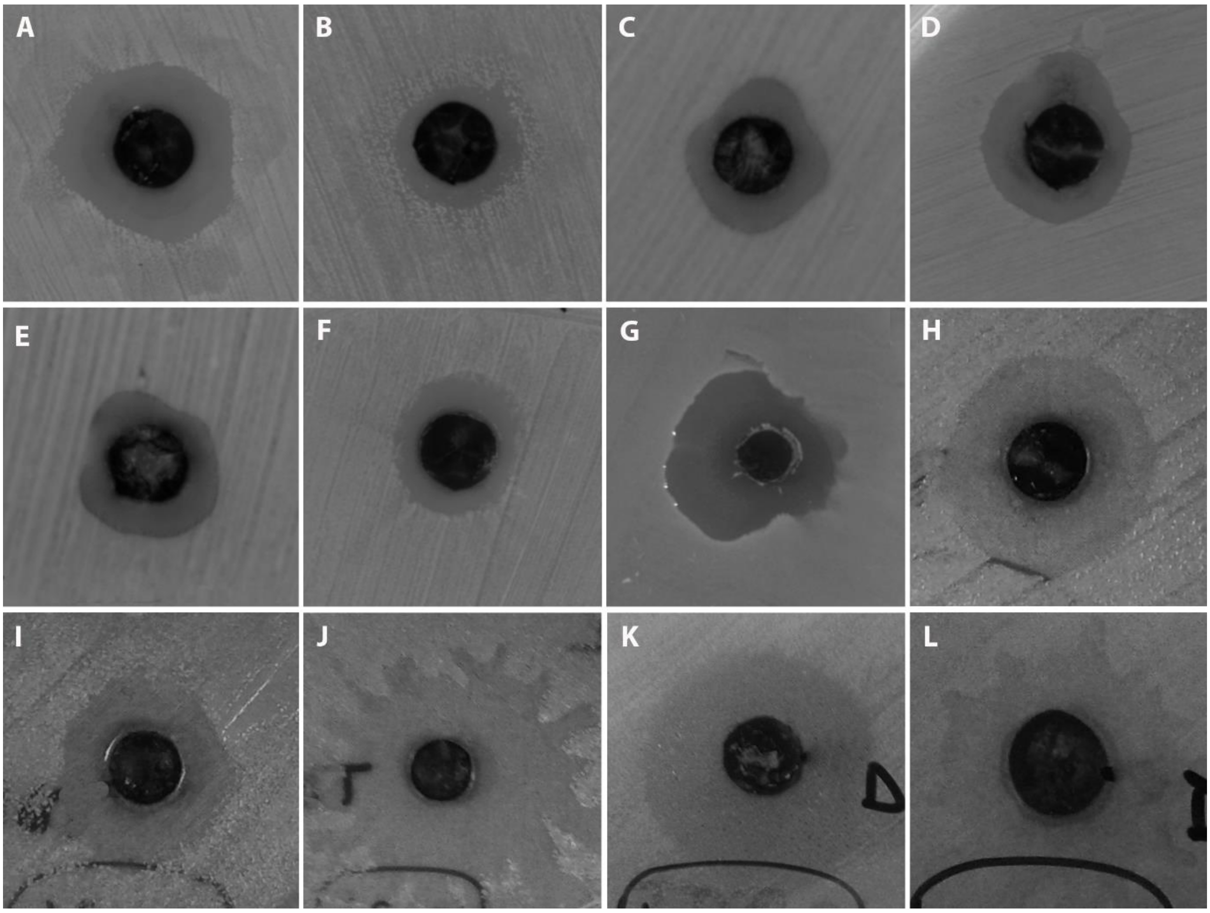

Suppl. Fig. 2 Example of antimicrobial effect (inhibition zones) of *J. lividum* ROICE173 on MDR bacteria. **A**: CE45 - *Enterococcus faecalis* (environmental isolate). **B**: CE58 - *Enterococcus faecium* (environmental isolate). **C**: EE6 - *Escherichia coli* (environmental isolate). **D**: CI - *Enterococcus facecium* (environmental isolate). **E**: EE16 - *Klebsiella oxytoca* (environmental isolate). **F**: CI23 - *Enterococcus qallinarum* (environmental isolate). **G**: CS5 - *Enterococcus faecium* (environmental isolate). **H**: EK0255 - *Staphylococcus aureus* (clinical isolate). **I**: EK8796 - *Staphylococcus aureus* (clinical isolate). **J**: EH9526 - *Staphylococcus aureus* (clinical isolate). **K**: EK7929 - *Staphylococcus hominis* (clinical isolate). **L**: EK9667 - *Enterococcus faecium vanA* (clinical isolate). Well diameter = 6 mm.

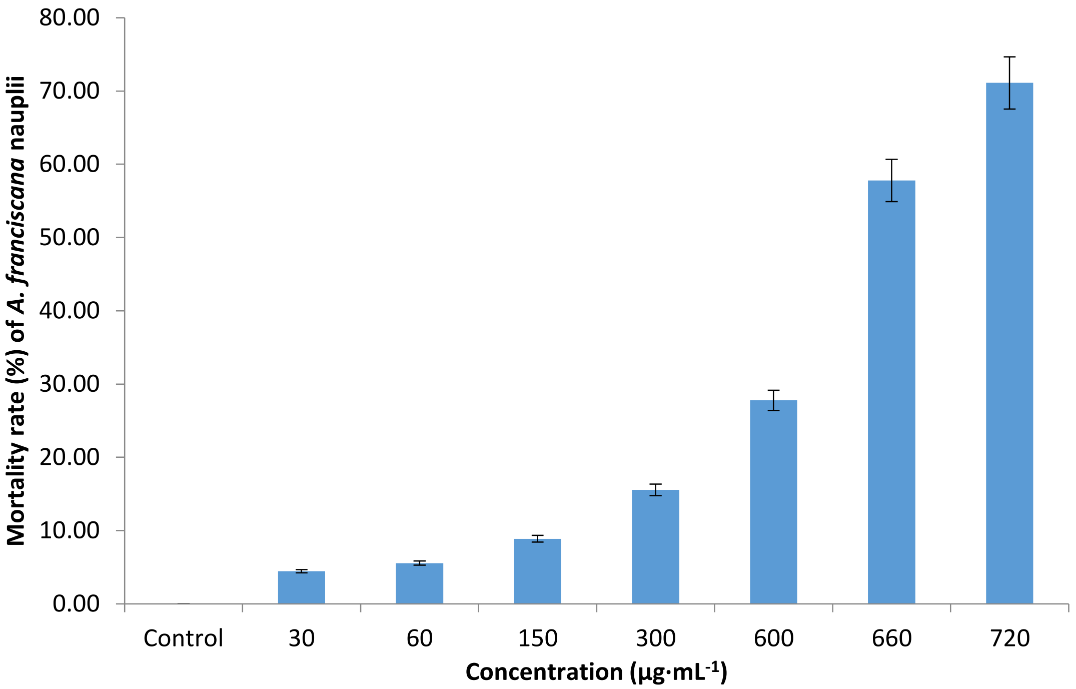

Suppl. Fig. 3. Mortality rate of *Artemia fransciscana* nauplii against the specified concentration of violacein in the *J. lividum* ROICE173 extract.
